# Structural brain complexity is associated with linguistic complexity in psychosis

**DOI:** 10.1192/j.eurpsy.2026.12242

**Published:** 2026-07-21

**Authors:** Alexandra Korda, Wolfram Hinzen, Rui He, Peter van Dyken, Michael Mackinley, Eric Toyota, Mihai Avram, Christina Andreou, Stefan Borgwardt, Lena Palaniyappan

**Affiliations:** 1 Translational Psychiatry, Department of Psychiatry and Psychotherapy, University of Lübeck, Germany; 2 Department of Translation & Language Sciences, Universitat Pompeu Fabra, Carrer Roc Boronat, Barcelona, Spain; 3 Institut Català de Recerca i Estudis Avançats (ICREA), Barcelona, Spain; 4 Robarts Research Institute, Schulich School of Medicine and Dentistry, Western University, London, Ontario, Canada; 5 Douglas Mental Health University Institute, Department of Psychiatry, McGill University, Montreal, Quebec, Canada; 6 Department of Medical Biophysics, Schulich School of Medicine and Dentistry, Western University, London, Ontario, Canada

**Keywords:** brain complexity, language models, linguistic complexity, NLP, psychosis

## Abstract

**Background:**

Brain-structural and language abnormalities in people with psychotic symptoms (PSx) have long been reported. A key question is whether language alterations are a potential behavioral readout of brain cortical gray matter changes. Adding to previous evidence for this in the cases of cortical thinning and gyrification, we here used structural MRI (sMRI) to relate a complexity-theoretical metric, the Largest Lyapunov Exponent (lambda [λ]), to computational linguistic complexity metrics extracted from spontaneous speech. This study aimed to identify psychosis-related regional changes in structural complexity using λ and an explainable AI (XAI) approach. We hypothesized that altered structural complexity will relate to computational language features that have previously been shown to track brain dysconnectivity in schizophrenia in fMRI.

**Methods:**

MRI data were acquired from 92 patients with PSx and 38 healthy controls (HCs) from the TOPSY study. Nonlinear analysis of gray matter distribution was performed by extracting the λ for gray matter voxels. XAI was employed to identify the voxels contributing significantly to classifying PSx against HCs. Finally, the brain voxels’ contribution was tested for associations with word perplexity and syntactic and semantic complexity metrics, from spontaneous speech as obtained from picture descriptions, and with clinical symptom severity (SOFAS and PANSS scores).

**Results:**

The classification framework resulted in a balanced accuracy of 75.9%. The voxels contributing most to the classification decision were located in the temporal lobe, cingulum, angular, lingual, calcarine, occipital, fusiform, and parietal cortices, and parts of the cerebellum and vermis. Structural complexity in these regions was significantly associated with clinical variables (SOFAS and PANSS), word perplexity, and lexical diversity.

**Conclusions:**

We present a new analytical framework using spatial series extracted from sMRI, demonstrating that alterations in structural complexity are an identifiable feature of psychosis, which co-vary with probabilistic and structural changes in speech as a behavioral readout.

## Introduction

In schizophrenia (SCZ), abnormalities in cortical surface morphology have long been noted. Even very early stages of this illness are associated with reduced cortical folding across multiple brain regions [1], especially those that are relevant to language [2]. These morphological changes have been largely studied using approaches that quantify the amount of “buried” versus outer cortex in cortical neighborhoods (local gyrification index), but this cannot be computed for subcortical structures, and fails to take into account how the structural complexity of each point of the brain varies in relation to the initial conditions of a developing brain that takes shape from a smaller mass. As a result, while altered gyrification has been linked to long-term prognostic outcomes (e.g., transition to psychosis [3], poor treatment response [4], and poor social functioning [5]), to date, regional folding changes have only been weakly associated with cognitive and behavioral differences in patients with psychotic symptoms (PSx) [6]. Maturational patterns, especially involving the subcortex [7], are critical determinants of poor cognitive and psychiatric outcomes. Comprehensive quantification of structural complexity in PSx requires approaches that capture geometric shape variations more holistically.

Analyses of nonlinear dynamics are an appropriate mathematical method for deriving meaningful statistical features from the intrinsic complexity of gray matter morphology (GM). An earlier work that converted MRI scans into spatial series of distances (between the center of the brain, as a mass, and the voxels across the brain) compared the Largest Lyapunov Exponent (lambda [λ]) values between patients and controls and revealed changes in the complexity of brain folding in first-episode psychosis (FEP), clinical-high risk (CHR), and SCZ [8]. λ is a geometric shape descriptor of cortical surface morphology, which also considers subcortical areas. It measures how sensitive a system is to initial conditions by measuring the average exponential rate of divergence from these conditions, using a distance metric between voxels. We can expect people with psychotic symptoms (PSx) to differ from healthy controls (HC) in the voxel-wise values of λ from the GM distribution, generating a PSx-related pattern of aberrant structural complexity that can be ascertained using an explainable artificial intelligence approach (XAI) [9]. The λ spatial series extracted from the voxels that contribute most to the separation of people with PSx from HC may influence key behavioral aspects of PSx.

A key question concerns evaluating possible behavioral readouts of altered brain cortical gray matter measurement and function in PSx. While such alterations are associated with both clinical symptom scores and neuropsychological tests [10], naturally produced speech has recently emerged as a promising behavioral marker in PSx, with numerous studies demonstrating that differences in acoustic-prosodic as well as semantic and structural linguistic measures are predictive of diagnosis, symptoms, or disease progression [11]. Spontaneous connected speech always requires multiple cognitive capacities interacting coherently, beyond linguistic knowledge, such as declarative memory of words and meanings, procedural memory as involved in syntax, executive functions as involved in speech and discourse planning, and social cognitive functions. By integrating cognition at large, language could be a subtle indicator of cognitive changes and disintegration, consistent with the view that language forms a common factor (“L-factor”) across mental disorders [12]. The likelihood of a direct brain structure/complexity and linguistic structure/complexity relationship is foretold by previous work linking altered linguistic behavior to cortical thinning [13] and gyrification [14], as well as brain-functional differences, such as effective connectivity in the semantic brain network [15], functional connectivity gradients [16], or neural self-inhibition [17].

In this analysis, we investigated (a) PSx-related regional changes in complexity using XAI and (b) the association of structural complexity in PSx with alterations in language production as measured through a comprehensive range of computational linguistic metrics across quantitative, syntactic, information-theoretic, and semantic feature domains. Our specific hypothesis was that, similar to what has been shown with brain-functional connectivity [16], language measures would be sensitive to alterations in the λ parameter.

## Material and methods

### Participants

We included patients with psychotic disorders – predominantly schizophrenia – across various stages, named as the PSx group. In this group, there were *n* = 60 with first-episode psychosis (FEP), of whom 49 had schizophrenia, 2 had bipolar disorder with psychosis, 2 had major depression with psychosis, 3 had schizoaffective disorder, and 4 had psychosis not otherwise specified. In addition, 16 patients had established schizophrenia with treatment exposure for at least 3 years (SCZ) and 16 were in clinical high risk (CHR), and these groups were compared to 38 healthy controls (HCs). FEP patients had to meet two requirements to be included: (1) those who have taken antipsychotic drugs for <2 weeks throughout their lifetime; and (2) be experiencing their first clinical manifestation of psychotic symptoms. The CHR group, recruited from a prodromal psychosis clinic (the PROSPECT program at London Health Sciences Center, London, Ontario), included only help-seeking subjects meeting Attenuated Psychosis Syndrome or brief and limited intermittent psychosis threshold as defined by Structured Interview for Psychosis-risk Syndromes; CHR subjects were included only if they had no prior lifetime antipsychotic exposure. Patients with established schizophrenia were included if they were treated for over 3 years since the onset of their illness and were clinically stable (on long-acting injectables, with no recorded hospitalizations in the past year and receiving community-based care). Three years was selected as the threshold for determining established schizophrenia as it reflects the transition out of the FEP phase and into routine care as guided by regional psychosis standards, and this ensured the established group was as close as possible in age to the FEP group. Healthy controls, group-matched for age, sex, and parental socioeconomic status, who had no personal history of mental illnesses and no family history of psychotic disorders, were included. All participants had no significant head injury, drug/alcohol dependence or major medical illnesses, were fluent in English, and provided written informed consent to participate in the study [18]. The demographic characteristics of the participants are presented in Table 1.

**Table 1.** Demographic and clinical variables Table 1. long description.

Variable	PSx Mean (std)	HC Mean (std)	FDR-corrected *p*-value
*N*	92	38	
Sex	0.19 (0.17)	0.32 (0.47)	0.17
Age	23.02 (4.79)	21.79 (3.51)	0.11
Education	12.71 (1.74)	14.14 (2.15)	<0.001*
Marital_status	1.99 (0.38)	1.95 (0.23)	0.44
Clinical global impression severity (CGIS)	4.36 (1.87)	N/A	**–**
Social and occupational functioning assessment scale (SOFAS)	47.74 (15.98)	81.82 (4.34)	<0.001*
Daily drug use (Antipsychotic)	2.29 (4.03)	–	**–**
Learning difficulties	1.41 (7.49)	0.41 (0.82)	0.29
Handedness	9.28 (5.15)	10.41 (3.22)	0.15
Alcohol (AUDIT)	2.77 (2.72)	2.75 (2.68)	0.14
Smoking index	6.02 (5.94)	–	**–**
Cannabis (CAST)	10.74 (6.17)	13.5 (10.61)	<0.001*
Positive and negative syndrome scale (PANSS–8)			
Positive	9.89 (4.63)	–	
Negative	6.35 (4.16)	–	
General	4.65 (2.52)	–	
Total	20.83 (2.52)	–	

* FDR_corrected p-values < 0.05.

Diagnosis of psychotic disorders was made using a Best Estimate Consensus procedure as outlined in Leckman [19] based on all available clinical information, including diagnostic clinical interviews, and confirmed via 6–12 months follow-up in each case. Trained research assistants administered three black-and-white pictures from the Thought Language Index (TLI) interview [20], based on the Thematic Apperception Task [21] with standard prompts to elicit a description (i.e., “describe the picture… as fully as you can”). Interviewers were instructed to refrain from interrupting or premature prompting, to maintain interviewee-only speech as much as possible. All study techniques were authorized by Western University’s Research Ethics Board, and participants gave written informed consent for the study (see Tracking Outcomes in Psychosis [TOPSY]; ClinicalTrials.gov Identifier: NCT02882204 for other study details).

### MRI data acquisition

We used a Siemens MAGNETOM 7T MRI (Erlangen, Germany) (8-channel transmit/32-channel receive, head-only, radiofrequency coil) at Western University’s Centre for Metabolic Mapping in London, Ontario. A T1-weighted isotropic MP2RAGE sequence was used with the following parameters: repetition time = 6,000 ms, time to echo = 2.83 ms, inversion time TI_1_ = 800 ms, TI_2_ = 2,700 ms, flip-angle 1 (α_1_) = 4°, flip-angle 2 (α_2_) = 5°, field of view = 350 mm × 263 mm × 350 mm, T_acq_ = 9 min 38 s, iPAT_PE_ = 3 and 6/8 partial k-space, slice thickness = 0.75 mm.

### MRI data preprocessing

Before data centralization, every image was examined visually, automatically defiled, and anonymized using software based on Freesurfer. The images were then segmented into GM, WM, and CSF maps using the open-source CAT12 toolbox (version r1155; CAT12), an extension of SPM12 (Statistical Parametric Mapping: The Analysis of Functional Brain Images, SPM12), and normalized to the stereotactic space of the Montreal Neurological Institute coordinates (MNI-152 space). Spatial filtering, segmentation, segmentation estimation, a local adaptive segmentation step (which corrects the images for WM homogeneities and different GM intensities), and a high-dimensional Diffeomorphic Anatomical Registration Through Exponentiated Lie Algebra (DARTEL) registration of the image to an MNI template in the IXI database (IXI) were the processing steps that were employed with the CAT12 toolbox. The processing procedures used on the structural pictures are described in full in the CAT12 toolbox handbook. The CAT12 toolbox’s established quality-assurance approach was used to assess the GM volume maps’ quality. If artifacts were found during the CAT12 quality-assurance framework, the images were examined and, if necessary, eliminated. There were no artifacts indicated in this study. For additional analysis, the modulated wrapped gray-matter segmented magnetic resonance images, or mwp1*images, were utilized.

### Feature extraction

#### Brain complexity

We calculated the Largest Lyapunov Exponent (λ) from the gray-matter segmented images using a spatial series extracted from the Euclidean distances between the center of the brain and each voxel, weighted by the voxel intensity. The first step was to calculate the center of mass of the modulated and normalized GM segments using the function named “center of Mass” in MATLAB R2016b. The distance of the GM center of the brain, weighted with the voxel intensities (distance in mm × voxel intensity), was calculated to enhance the weight of the short-distance voxels compared to the long-distance voxels by including the intensity of the voxels. In that way, we capture geometric changes in the whole brain as a function of weighted voxel distance (WD). To reduce the computational complexity, we sorted (from higher to lower) the WD of all brain voxels and selected 10,000 voxels with the highest WD (more details in previous studies [8]). Then, these distances were used as a spatial series to reconstruct the matrix of the state space and to estimate the λ value [22, 23]. These values indicate the dependency of the dynamical gray matter space on the initial conditions, that is, how a small starting difference can lead to larger transitions over space. High positive lambda values mean greater complexity (chaos; dependency on initial conditions) in that region.

**Figure 1.**
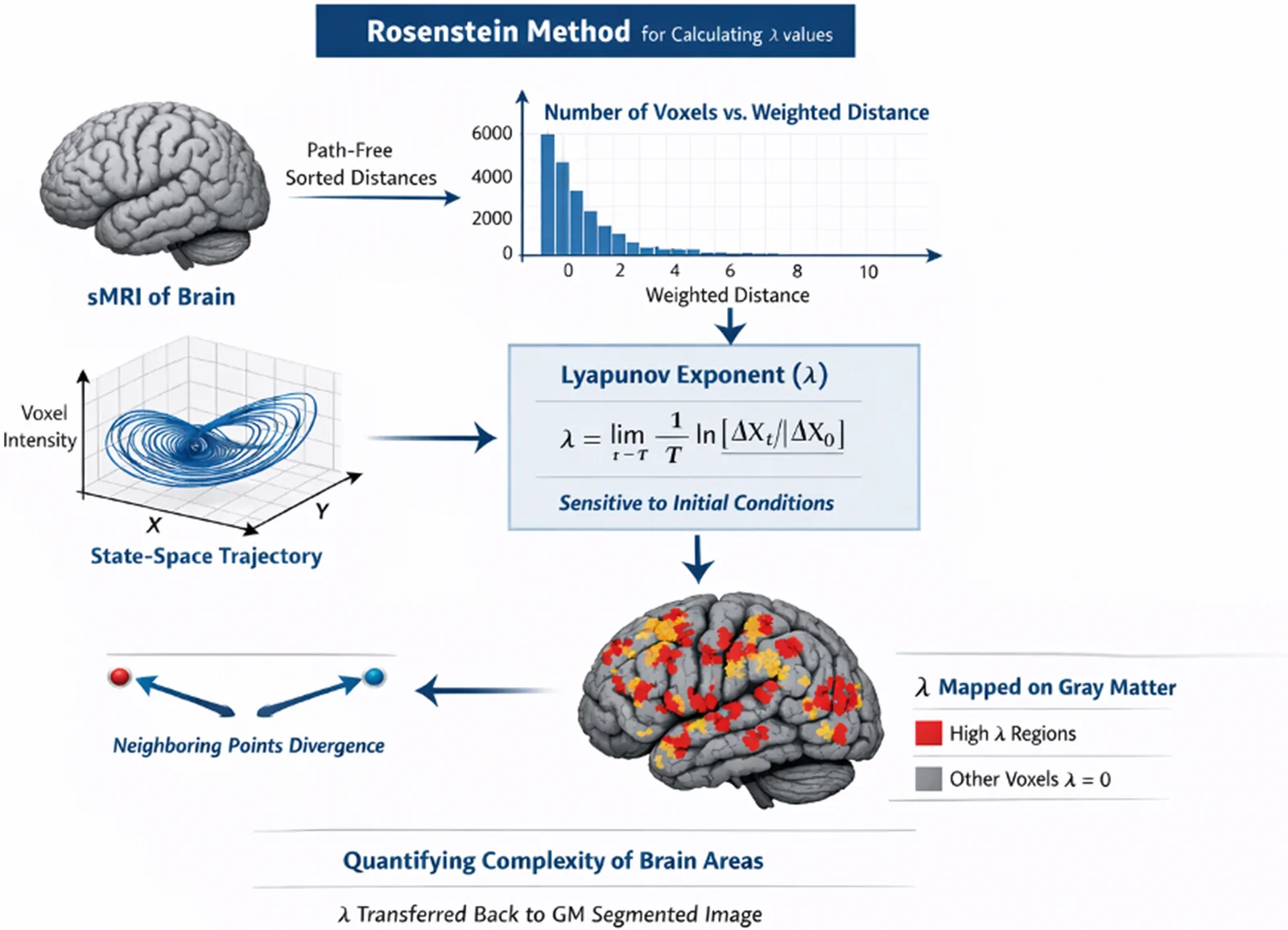
Workflow for the calculation of λ on sMRI data. Figure 1. long description.

To determine these λ values, a method created by Rosenstein was used [24], which is robust to noise, reconstruction delay, and embedding dimension. In brief, the spatial series of the path-free sorted distances taken from the brain’s sMRI can be represented by a dynamical system, in which the distance from the center of the brain and the voxel intensity are substituted for the parameter space. The sensitive dependency of this dynamical system on initial conditions is then represented by positive λ values. Takens’ Theorem [25] describes the trajectory of a system’s attractor (i.e., the pattern that the system tends to settle around, which changes with geographic location over the state-space) and anticipates the interactions between different voxel intensities. The collective interactions across locations between voxel intensities can be used to quantify the complexity of brain areas, using the stored coordinates of the WD of each voxel. λ values were transferred back to GM segmented images for each individual. Voxels that were not chosen as the top-weighted voxels had their λ values adjusted to 0. The method of the λ calculation captures how two neighboring points in the state-space (phase space) diverge over the surface with respect to all the other distance points in the brain. Consequently, λ permits not only the identification of abnormalities in the gray matter but also how these spread across the brain. Unlike static morphometric measures, λ captures the sensitivity of spatial structural organization to local perturbations, reflecting distributed morphological coordination. More details for the calculation of λ are included in the Supplementary Material. We additionally ran a Levene test to compare the values of λ between groups and present the z-scores of λ per group. Figure 1 presents the workflow of the calculation of the brain complexity.

#### Speech feature extraction

**Figure 2.**
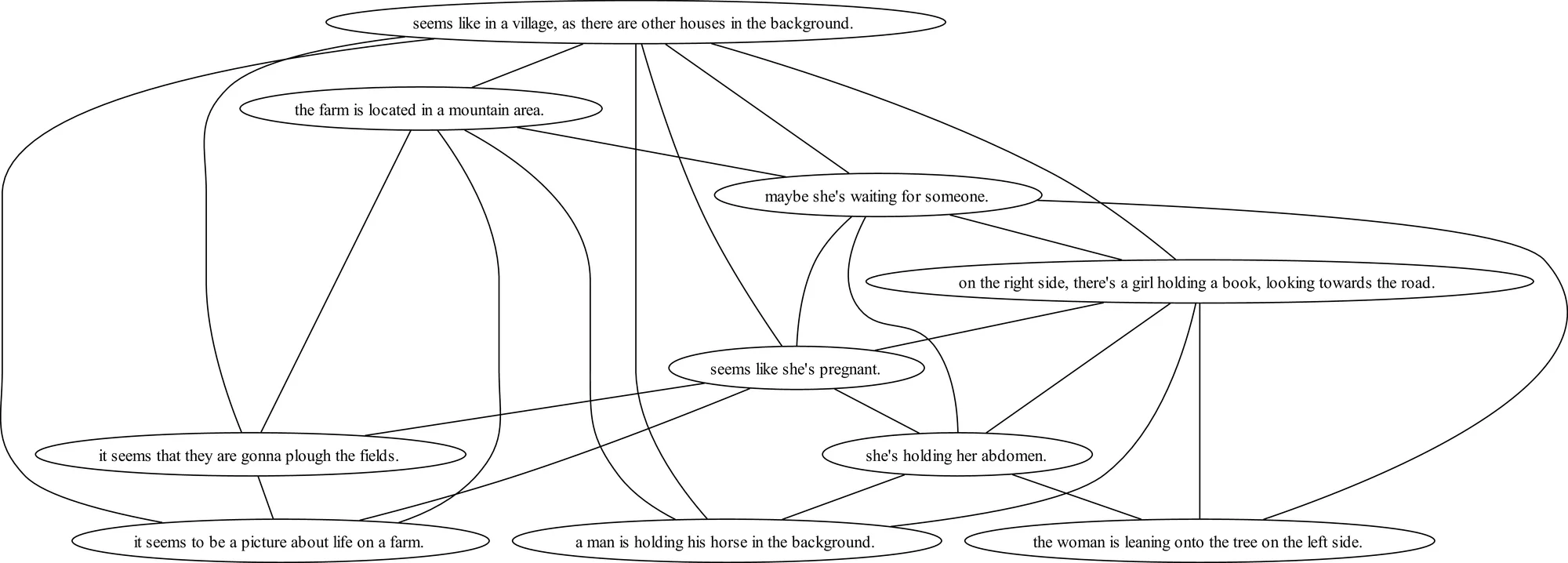
An example of a graph built from tokens by thresholding a semantic similarity matrix. Each node is a linguistic unit (sentence in this example), and the edges are the semantic similarities remaining after thresholding. Figure 2. long description.

One-minute speech samples were elicited from each picture through audio recordings, which were transcribed by graduate-level research assistants. All lowercase sentences were created from the transcriptions, and manual judgment was used to define the utterance borders. Interviewer segments, as well as participants’ nonspeech data (such as laughing or fillers like “uhm”), were manually removed, leaving patient-only speech data to be analyzed. All speech transcripts were manually segmented into utterances. Our selection of variables to be extracted was driven by an aim of comprehensiveness across different linguistic levels of analysis (quantitative, syntax, and semantics) and previous knowledge of linguistic metrics showing sensitivity to psychosis [15, 16, 26]. We calculated six broad quantitative features that describe the quantitative speech features: the number of sentences (SentNum), the number of words (WordNum), the average sentence length (SentLen), the number of lexical categories (defined as nouns, verbs, and adjectives, LexCatNum), the type-token ratio of words (WordTTR), and the type-token ratio of lexical categories (LexCatNum). To obtain more specific measures of syntactic complexity, every utterance was parsed into a constituency tree in Chomsky Normal Form indicating its internal phrasal structure – how words form phrases and finally utterances. From these trees, we computed the ratio of a particular phrasal node count by the total node counts (Phrases), as well as the depth of the tree (Depth), defined as the maximum number of edges that need to be traversed to reach from the source node (sentence node) to the furthest target node (token node, forming the leaves of the tree). For semantic complexity, we investigated the structure of semantic space at three different levels: (i) individual lexical categories using the English fastText model, (ii) contextualized subwords using a BERT model [27] (Bert-base-uncased), and (iii) sentences using a sentence transformer (sentence-transformers/all-MiniLM-L6-v2). At each of these levels, we first analyzed the averaged local semantic similarity (LSim) between the embeddings of consecutive linguistic units (lexical categories, contextualized subwords, or sentences), and the similarity between each unit and the centroid of the embeddings (CSim), where the centroid is an approximation of the overall topic of the speech sample. In a next step, and for a finer-grained categorization exploiting the topology of the semantic space, we computed semantic similarity using the normalized cosine similarity scores between every pair of two linguistic units (i.e., content words for fastText, subtokens for BERT, or sentences for SBERT). The semantic similarity matrices were then binarized by proportional thresholding. The threshold was picked dynamically following previous work [16]. We selected the lowest threshold from 0.05 to 0.8 with intervals of 0.05, which returns a sparsified matrix with the average degree (the degree of a node is the number of connections linked to the node) over all nodes larger than 2 multiplied by the e-base logarithm of the number of nodes (2log*N*) [26] (see Figure 2). We then characterized the topology of each semantic graph in terms of global integration and local segregation, using closeness centrality (CC) and clustering coefficient, respectively. For each graph, both measures were first computed at the node level and then averaged across all nodes to obtain graph-level summary values. Furthermore, we compared the similarity between the speech and prompt picture using a bimodal (visual–language) model (CLIP). Finally, we extracted perplexity, an information-theoretic measure, using Mistral (Word_PPL). For all linguistic features, measurements were first computed separately for each individual one-minute speech sample elicited by each of the picture prompts. Participant-level values were then obtained by averaging the corresponding sample-level measures across the three available samples within each participant. Statistically significant differences between PSx and HC, using one-way ANOVA (Welch’s), were presented for most of the speech features extracted (see Table 2 and Supplementary File).

**Table 2.** Comparison of linguistic features across groups Table 2. long description.

Variable	PSxMean (std)	HCMean (std)	FDR-corrected *p*-value
SentNum (number of sentences)	11.15 (3.14)	12.7 (2.58)	0.02*
SentLen (length of the sentences)	11.42 (2.01)	11.93 (1.99)	0.19
WordNum (number of words)	122.96 (36.36)	143.26 (29.8)	0.001*
WordTTR (the type-token ratio of words)	0.88 (0.09)	0.86 (0.16)	0.47
LexCatNum (the number of lexical categories)	462 (237)	583.6 (246.3)	0.012*
LexCatTTR(the type-token ratio of lexical categories)	0.72 (0.16)	0.72 (0.12)	0.95
Phrases	1.21 (0.05)	1.19 (0.044)	0.004*
Depth	8.65 (1.35)	8.80 (1.21)	0.54
Word_PPL (perplexity)	6.95 (1.82)	5.63 (0.89)	<0.001*
CLIP (similarity between the speech and prompt picture)	0.17 (0.012)	0.18 (0.012)	0.004*
Fast text model			
LSim (local semantic similarity)	0.24 (0.03)	0.23 (0.02)	0.03*
CSim (the similarity between each unit and the centroid of the embeddings)	0.47 (0.03)	0.47 (0.02)	0.047*
CC (closeness centrality)	0.49 (0.05)	0.46 (0.03)	<0.001*
Cluster	0.49 (0.05)	0.49 (0.04)	0.39
Sentence transformers			
LSim (local semantic similarity)	0.71 (0.11)	0.73 (0.02)	0.03*
CSim (the similarity between each unit and the centroid of the embeddings)	0.83 (0.12)	0.86 (0.01)	0.02*
CC (closeness centrality)	0.65 (0.09)	0.66 (0.04)	0.76
Cluster	0.62 (0.12)	0.63 (0.08)	0.45
BERT			
LSim (local semantic similarity)	0.49 (0.07)	0.51 (0.02)	0.003*
CSim (the similarity between each unit and the centroid of the embeddings)	0.55 (0.08)	0.58 (0.01)	0.008*
CC (closeness centrality)	0.36 (0.05)	0.37 (0.02)	0.51
Cluster	0.42 (0.06)	0.43 (0.02)	0.043*

* FDR_corrected p-values < 0.05.

### Classification framework-evaluation of the classifier

**Figure 3.**
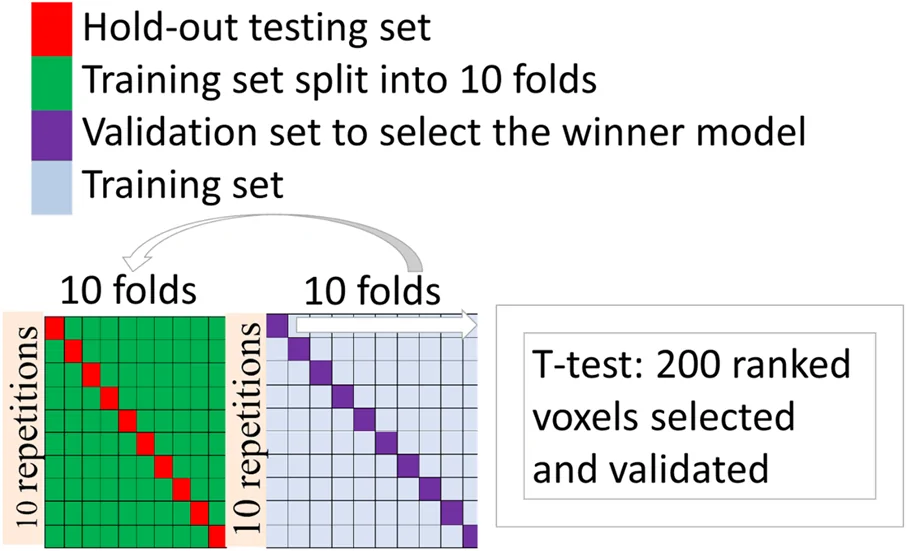
The classification framework: a 10-times repeated 10 × 10 nested CV approach. Figure 3. long description.

To discriminate PSx psychopathology against HC, we pooled the λ feature maps from all clinical groups. We loaded the λ feature maps into a multilayer neural network in MATLAB R2016b using 10 times repeated 10 × 10 nested cross-validation approach. The inner cycle’s voxel selection (two-sample *t*-test) was cross-validated by choosing a subset of features that were relevant to the database’s dimension, specifically the top 200 ranked voxels, as expressed by the λ values, that best distinguished between the two classes (see Figure 3).

For localization of the identified brain changes in PSx versus HC participants, we calculated the relevance of the voxels in each class using the layer-wise relevance propagation (LRP) algorithm for multilayer neural networks, as described by Bach and co-workers [28]. The output of the algorithm is an individual heatmap that represents common changes in brain cortical gray matter measurements for PSx. The final images were visualized using the MRIcron toolbox (MRIcron; v1.020190902 for Windows) using the aal.nii (Automated Anatomical Labeling). Visualizations of the classification results on the holdout dataset are presented in Figure 2. The regions of interest were extracted using the AAL-VOIs atlas (AAL-VOIs).

### Associations of brain complexity with clinical variables

Using the LRP algorithm, an individual heatmap is produced for each correctly classified subject in PSx. The contribution of the voxels produced by the LRP algorithm was then associated with clinical variables (SOFAS and PANSS scores) and linguistic features (quantitative, syntactic, and semantic complexity, and an information-theoretic measure: perplexity). Specifically, we performed partial correlation analysis using age, sex, education, medication, and group label to investigate the association of brain complexity with clinical symptoms and linguistic measures from picture descriptions [21] (adding SentLen as a covariate).

## Results

### Clinical evaluations

Sociodemographic and clinical characteristics of the PSx group and HC are presented in Table 1. Statistically significant differences using one-way ANOVA (Welch’s) were presented for most of the clinical scores except learning difficulties, handedness, alcohol use, age, sex, and marital status.

### Calculation of λ values

**Figure 4.**
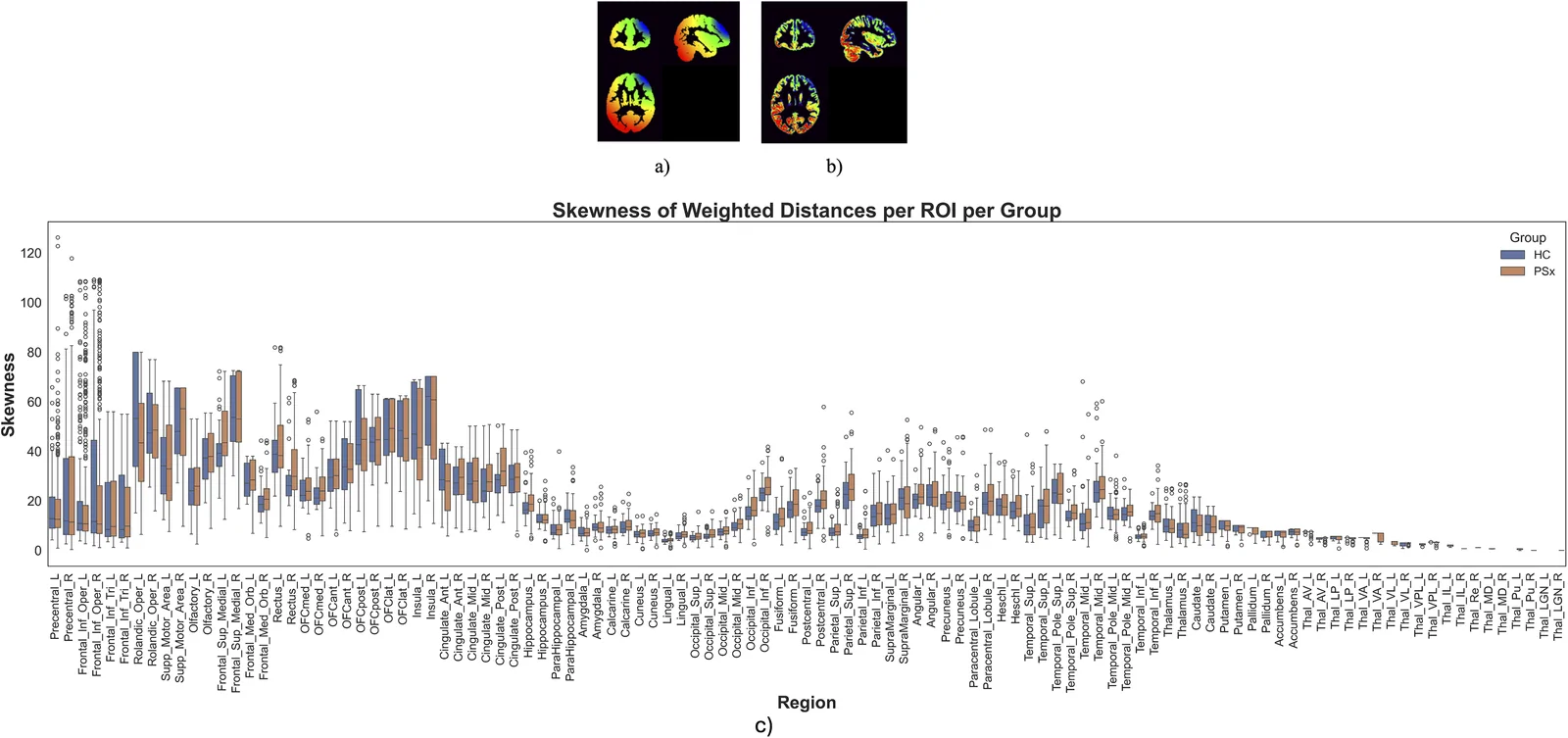
(A) The distances between the center of mass and all brain voxels, (B) the weighted distances (multiplied by the intensity of the voxels) between the voxels and the center of mass, and (C) mean and standard deviation of the skewness of the weighted distances per region per group. Figure 4. long description.

Top-weighted distances correspond to voxels that either belong to the cortical surface or are slightly close to the center of mass of the GM. For example, one point with intensity 1 and distance from the GM center of mass 3 has the same weighted distance as one point with intensity 3 and distance 1. It is observed that the highest distances are located in the temporal and occipital lobes (red color in Figure 4A). However, high weighted distances are also located at the middle frontal gyrus, precuneus, left parietal, and left caudate (Figure 4B). To address concerns about skewed distributions, we visualized the skewnesses of the weighted distances per region per group in Figure 4C. While the distribution of the weighted distances is slightly skewed, the majority of values are well distributed across the selected voxels (see Figure 4C).

**Figure 5.**
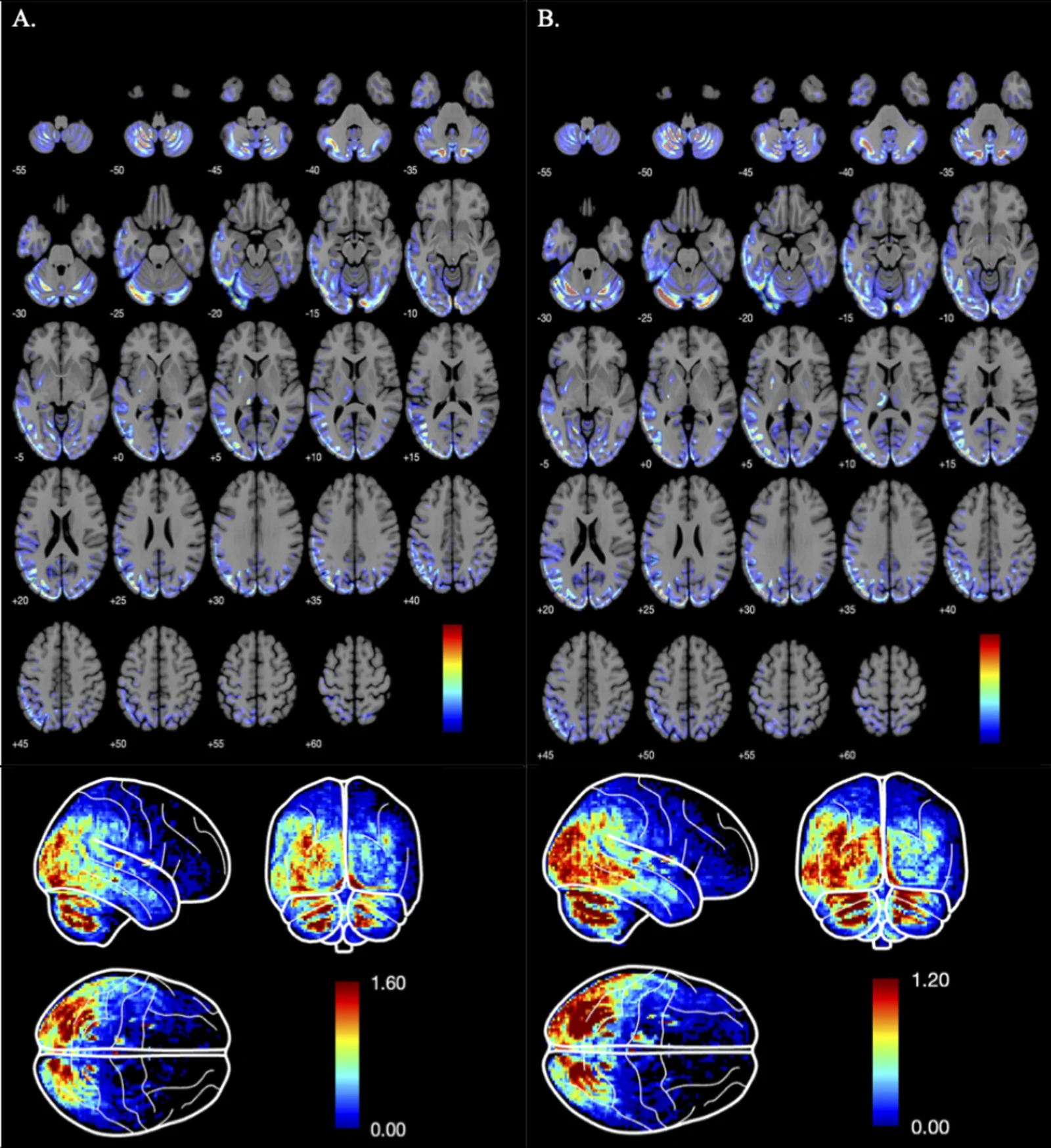
The mean λ values for the (A) HC group and the (B) PSx group. The color bar represents the range of the values of λ. Figure 5. long description.

**Figure 6.**
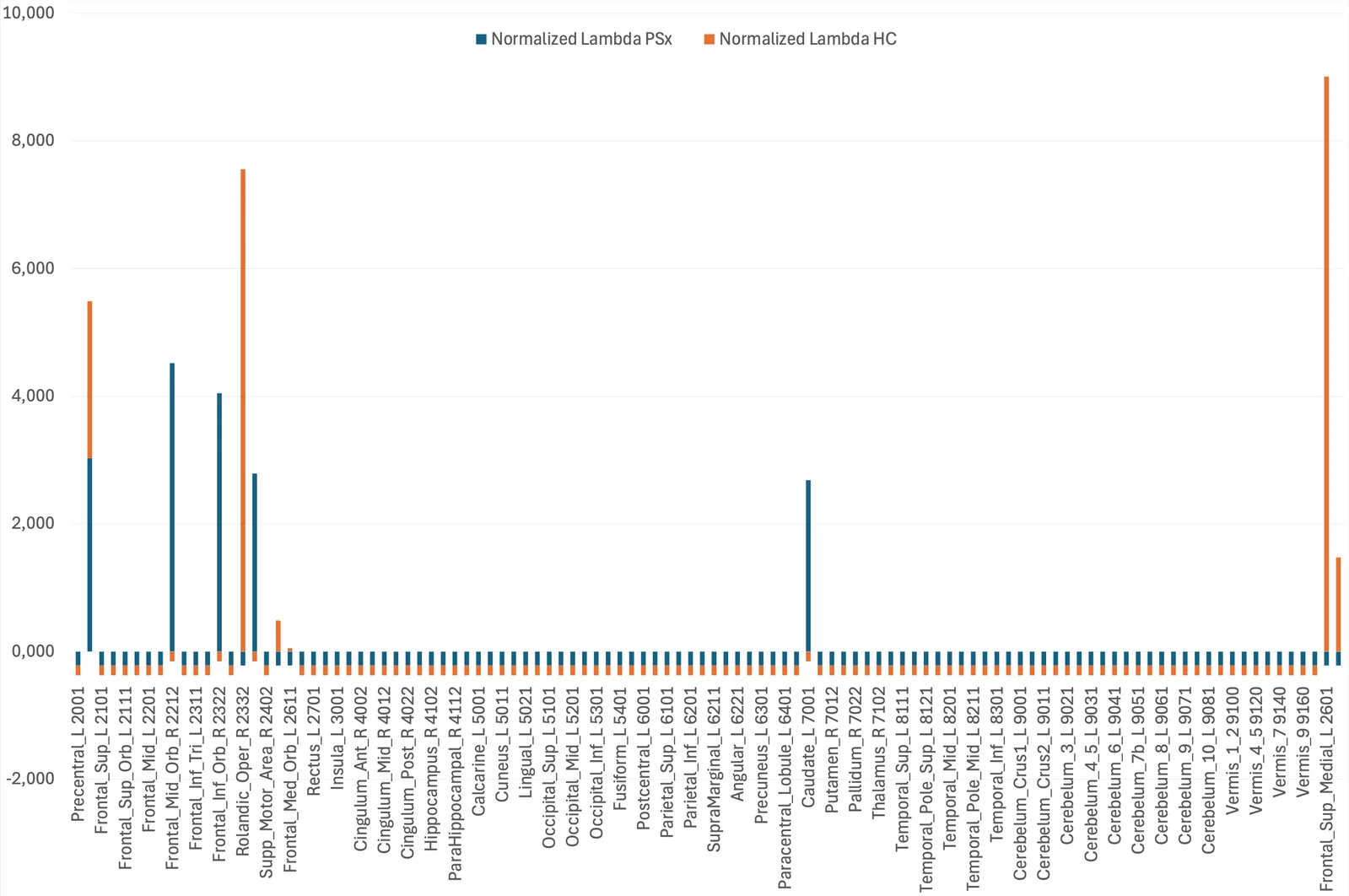
Z-score of structural complexity (lambda) values between groups per region for the selected voxels. Figure 6. long description.

Due to the selection of the 10,000 voxels using the criterion of the weighting distance, most of the voxels are located at the posterior brain regions (Figure 5). The mean λ values for the HC and PSx groups are presented in Figure 5. The regions that contributed significantly to the classification decision are presented in Table 3. For potential non-normality of the λ distribution, we calculated the z-score of the lambda values per group in the selected regions, as presented in Figure 6. Quantitatively, the mean ± std of the λ was 1.88 ± 0.13 for controls and 1.88 ± 0.14 for PSx, without a significant difference in variability between groups (Levene’s test, *p* = 0.38, Figure 6). Therefore, the key biological insight provided by the structural complexity measure does not arise from differences in variability, but rather from feature-specific patterns across the brain. For example, certain brain regions show consistent increases or decreases of structural complexity in the patient group compared with controls, highlighting potentially meaningful alterations in neurophysiological function captured by this novel measure.

**Table 3.** Associations of λ values from the voxels that contribute positively to classification decisions based on the LRP values (brain regions indicated by the aal.nii atlas) with clinical symptoms and linguistic variables Table 3. long description.

Brain regions	PANSSGlobal	PANSSNegative	PANSSPositive	SOFAS	Word_PPL	Word_TTR
Calcarine_L	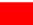	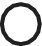	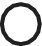	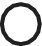	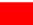	
Cuneus_R	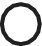	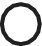	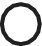	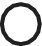	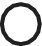	
Lingual_L	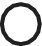	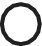	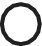	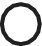	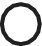	
Occipital_Mid_L	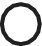	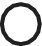	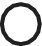	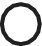	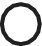	
Occipital_Inf_L	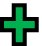	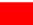	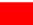	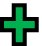	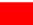	
Occipital_Inf_R	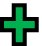	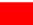	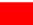	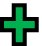	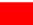	
Fusiform_L	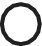	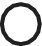	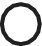	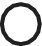	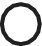	
Temporal_Mid_L	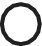	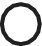	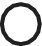	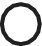	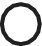	
Temporal_Inf_L	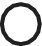	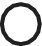	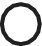	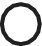	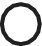	
Cerebellum_Crus1_L	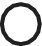	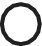	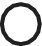	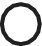	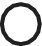	
Cerebellum_Crus1_R	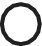	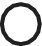	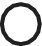	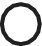	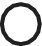	
Cerebellum_Crus2_L	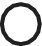	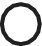	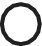	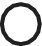	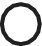	
Cerebellum_Crus2_R						
Cerebellum_6_L	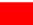	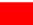	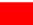	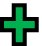	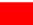	
Cerebellum_8_R	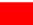	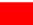	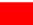	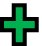	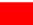	
Cerebellum_9_L	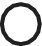	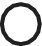	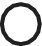	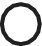	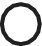	
Cerebellum_9_R	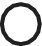	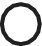	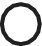	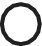	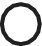	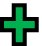
Cingulum_Post_L	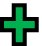	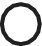	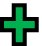	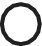	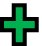	
Calcarine_R	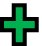	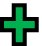	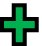	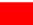	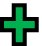	
Cuneus_L	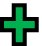	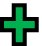	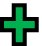	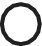	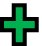	
Lingual_R	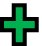	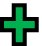	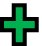	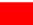	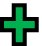	
Occipital_Sup_L	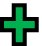	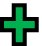	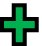	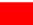	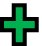	
Occipital_Sup_R	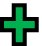	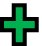	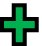	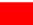	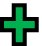	
Occipital_Mid_R	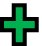	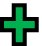	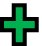	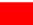	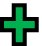	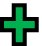
SupraMarginal_L	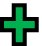	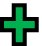	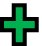	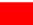	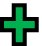	
Angular_L	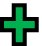	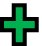	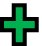	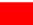	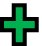	
Precuneus_L	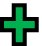	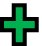	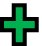	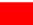	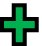	
Temporal_Sup_L	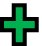	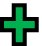	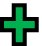	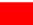	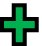	
Temporal_Mid_R	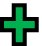	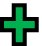	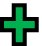	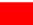	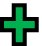	
Cerebellum_6_R	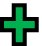	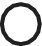	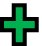	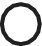	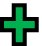	
Cerebellum_7b_L	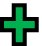	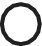	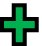	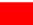	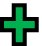	
Cerebelum_8_L	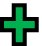	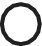	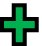	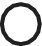	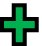	
Vermis_8	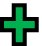	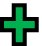	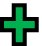	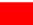	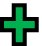	
Vermis_9	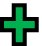	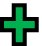	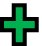	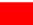		
Angular_R						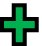

0: positive and negative voxels in the region, +: positive voxels, −: negative voxels and empty cell indicates no associations.

*Note*: Positive and negative associations are marked with plus and minus, respectively, while circles indicate regions with both positively and negatively associated voxels.

### Classification results and localization

**Figure 7.**
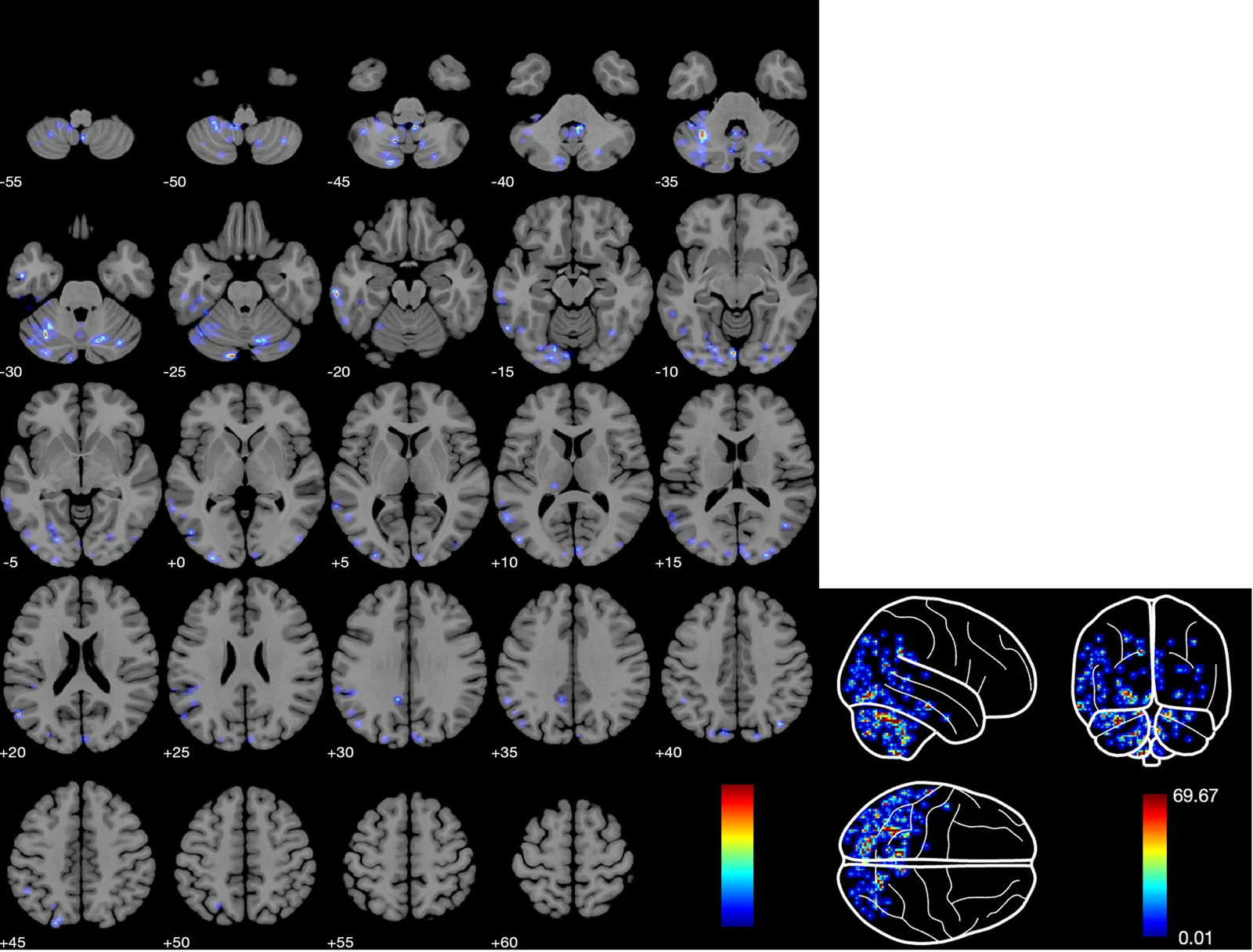
Significance of the contribution of each voxel to the classification decision for PSx versus HC calculated using the LRP algorithm. Figure 7. long description.

A repeated nested pooled cross-validation classifier of brain λ feature maps achieved a balanced accuracy of 75.9%, a sensitivity of 83.5%, and a specificity of 66.9% for discrimination of PSx versus HC participants. Common regions in PSx and HC subjects were selected by using the top 10,000 WD for each subject. The voxels that contributed significantly to the identification of the PSx group were located in temporal, occipital, lingual, calcarine, angular, parts of the cerebellum, and vermis (Figure 7 and Table 3).

### Group comparison of linguistic features

The groups were distinguishable linguistically by quantitative measures, specifically the number of sentences (SentNum), words (WordNum), phrases, and lexical categories (LexCatNum), as well as in the probabilistic measure of word perplexity (WordPPL). Semantic similarity metrics also distinguished the groups, as reported previously [26] for a subsample of the present dataset, in terms of similarity between narrations and pictures (using the bimodal model CLIP), as well as unimodal similarity using all language models, in both the local (FT_LSIM, Sent_LSim, and BERT_LSim) and centroid-based semantic similarity metrics (FT_CSim, Sent_CSim, and BERT_CSim). Additionally, in terms of semantic graphs, the FastText model differentiated groups in the closeness centrality (FT_CC), and the BERT model in the cluster coefficient (BERT_Cluster).

### Brain relevance association with clinical symptoms and linguistic metrics

Partial correlation analyses with age, sex, education, medication, sentence length, and group as covariates (FEP, CHR, and SCZ) showed associations between brain complexity, clinical and linguistic scores. The associations with FDR_corrected (FDR < 0.05) are presented in Table 3.

## Discussion

This study confirmed that gray matter abnormalities distinguish a group of patients with psychotic symptoms in comparison to HC, using the λ value as a novel complexity-theoretic metric applied to spatial voxel series. Moreover, the λ values in specific brain regions were associated with the probabilistic structure of speech, and (to a lesser extent) lexical richness. The method’s primary benefit is that it only needs 1% of the voxels in GM images; for low-complexity analysis, 10,000 voxels near the mass’s GM center or surface were enough to identify differences in brain topology in PSx against HC. Our intention was not to search for the best model that discriminates the two groups but to identify the voxels that can best differentiate PSx versus HC. Due to computational power, we selected 10,000 voxels to be analyzed, using the sorted weighted distances. By nature, the higher-distanced voxels in the brain from the center of mass are located at posterior brain regions. This is a limitation in our study due to methodological constraints.

Common brain abnormalities in CHR and FEP have been found in a previous study [8], the λ values in the temporal pole, right posterior cingulate gyrus, lingual gyrus, and fusiform in FEP significantly differentiated this group from HC. In line with the present study, PSx and HC showed statistically significant differences in the occipital and temporal lobes and cerebellum, which have been noted as indicators of psychotic illnesses before. The occipital and temporal lobes are implicated in PSx in numerous studies^13^: GM-intensity values in the occipital cortex were lower in subjects with a preponderance of attenuated psychotic symptoms [29]. Moreover, a thorough meta-analysis [30] found that morphological parameters taken from the temporal cortex in schizophrenia showed substantial variability. The observed between-group differences in the cerebellum are an important finding, as the cerebellum is part of the cortico-cerebello-thalamo-cortical circuit that has been related to disorganization of thought and language in schizophrenia [31]. The correlations between cerebellar region structural complexity values and perplexity illustrate the involvement of the cerebellum in higher-order cognitive processes, which is in line with previous studies. The cerebellum may affect how perceptual events are expressed lexically, which leads to poor thinking in early psychosis [32].

We observed mainly higher brain complexity in brain in the HC group compared to the PSx group, in line with a previous study [8]. Brain complexity was related to variables assessing speech, at various levels of analysis, particularly in the case of the information-theoretic metric of perplexity, which was previously linked to brain-functional anomalies in the cortex, using dynamical causal modeling with fMRI [15]. In this study, different pretrained language models, ultimately addressing different theoretical hypotheses in PSx, resulting in different sensitivity in PSx in this study. Remarkably, these associations were observed not only for occipital and temporal cortical regions, but also for key regions of the default mode network as well, such as the inferior parietal and posterior medial regions. In addition, extensive associations were seen in the cerebellum, where it is becoming increasingly clear how the cerebellum contributes to lexico-semantic comprehension [33, 34], as well as predictions, in both motor and cognitive domains, including language [35–37]. Perplexity is related to the surprise caused by a word in light of a contextual prediction. With cerebellar impairment frequently leading to subtle but higher-order speech and language issues, the cerebellum is now more broadly acknowledged as a component of the “extended language network” [35].

Regarding the clinical symptom associations, different parts of the cerebellum were associated negatively with PANSS scores and positively with SOFAS in the PSx group, replicating existing results in psychosis [38]. On the other hand, calcarine, cuneus/precuneus, lingual, angular_L, temporal, and occipital were positively associated with PANSS_scores and negatively with SOFAS. These brain regions have been considered as main neuroimaging markers for psychosis in studies using different metrics, such as texture features [10, 39] and conventional volumetric studies [40, 41]. Clinically, this indicates that structural brain differences are not merely anatomical but manifest in observable cognitive-linguistic behavior, providing a potential biomarker that bridges brain and symptom domains.

While in general there seems to be an accentuation toward the left hemisphere, interestingly, the right angular region is considered to act as a hub for modulation of language-related cortex [42], and in our study was only associated (positively) with Word_TTR. The angular gyrus, bilaterally, plays an important role in multimodal semantic cognition [43]. Word_TTR was also positively related to other regions in the right hemisphere, such as the middle occipital cortex and cerebellum_9. The role of the right hemisphere in the apprehension of complex language has been established [44, 45], and it is revealed in our study as well.

From a therapeutic and prognostic standpoint, these results suggest that geometric measures of gray matter organization could serve as early indicators of cognitive-linguistic vulnerability, guiding clinicians toward more refined prognostic assessments. By linking structural complexity to behaviorally meaningful outcomes, this approach provides a biologically grounded framework for interpreting patients’ cognitive and linguistic deficits, informing both clinical assessment and potential personalized treatment strategies.

Overall, integrating structural brain complexity with behavioral metrics offers a clinically interpretable bridge between neurobiology and observable symptoms, highlighting regions and networks that may drive key features of psychosis and informing future translational and therapeutic research.

## Limitations

To establish construct validity, λ should be evaluated against established morphometric measures and tested for incremental predictive value in clinical models. In the present analysis, patient groups were collapsed across diagnosis and stage of illness. While this approach allowed for larger sample sizes per analysis, it may have introduced additional heterogeneity and masked group-specific patterns. Future work should aim to stratify patients by diagnosis and illness stage to better capture potential differences in semantic organization across clinical subgroups. λ serves primarily as a complexity-theoretic metric reflecting current structural organization in adult brains instead of capturing “initial conditions” of brain development and direct evidence of historical maturational dynamics. Another limitation of the study concerns the effect of the antipsychotic treatment received by the SCZ group. While controlling for medication-related variance, we acknowledge that residual effects of cumulative exposure cannot be entirely ruled out and should be considered when interpreting the findings.

## Conclusion

The lambda parameter is a novel complexity-theoretic metric in the study of gray matter abnormalities and how these spread across the brain in spatial series. This new metric has here been confirmed to successfully distinguish between a mixed PSx sample and healthy controls, as well as relating to both clinical symptoms and computational metrics extracted from spontaneous speech. This newly reveals that spontaneous speech patterns, particularly the information-theoretic metric of perplexity, act as a read-out of brain-structural as well as brain-functional changes, with a particularly notable role played in this respect by the cerebellum.

## Supporting information

10.1192/j.eurpsy.2026.12242.sm001 Korda et al. supplementary material Korda et al. supplementary material

## Data Availability

Data are available upon written request to W.H. and L.P.

## Supplementary material

The supplementary material for this article can be found at http://doi.org/10.1192/j.eurpsy.2026.12242.

## Long descriptions

### Table 1 Long description

The table consists of four columns: Variable, P S x Mean (std), H C Mean (std), and F D R-corrected p-value.

Key data points include:

* N: P S x = 92, H C = 38.

* Sex: P S x = 0.19 (0.17), H C = 0.32 (0.47), p = 0.17.

* Age: P S x = 23.02 (4.79), H C = 21.79 (3.51), p = 0.11.

* Education: P S x = 12.71 (1.74), H C = 14.14 (2.15), p < 0.001.

* Marital status: P S x = 1.99 (0.38), H C = 1.95 (0.23), p = 0.44.

* Clinical global impression severity (C G I S): P S x = 4.36 (1.87), H C = N/A.

* Social and occupational functioning assessment scale (S O F A S): P S x = 47.74 (15.98), H C = 81.82 (4.34), p < 0.001.

* Daily drug use (Antipsychotic): P S x = 2.29 (4.03), H C = dash.

* Learning difficulties: P S x = 1.41 (7.49), H C = 0.41 (0.82), p = 0.29.

* Handedness: P S x = 9.28 (5.15), H C = 10.41 (3.22), p = 0.15.

* Alcohol (A U D I T): P S x = 2.77 (2.72), H C = 2.75 (2.68), p = 0.14.

* Smoking index: P S x = 6.02 (5.94), H C = dash.

* Cannabis (C A S T): P S x = 10.74 (6.17), H C = 13.5 (10.61), p < 0.001.

Positive and negative syndrome scale (P A N S S 8) for P S x group:

* Positive: 9.89 (4.63).

* Negative: 6.35 (4.16).

* General: 4.65 (2.52).

* Total: 20.83 (2.52).

Navigate back to Table 1.

### Figure 1 Long description

The flowchart is titled Rosenstein Method for Calculating lambda values.

* Top Left: An anatomical s M R I of Brain leads via an arrow labeled Path-Free Sorted Distances to a bar chart.

* Top Right: The bar chart titled Number of Voxels vs. Weighted Distance shows a right-skewed distribution. The y-axis ranges from 0 to 6000, and the x-axis for Weighted Distance ranges from 0 to 10.

* Middle Left: A three-dimensional plot titled State-Space Trajectory shows a blue spiral butterfly-shaped curve. The axes are Voxel Intensity, X, and Y.

* Center: Arrows from the bar chart and the state-space plot converge on a box titled Lyapunov Exponent (lambda). It contains the formula: lambda equals the limit as t approaches T of 1 over T times the natural log of the absolute value of Delta X sub t over Delta X sub 0. A note below states Sensitive to Initial Conditions.

* Bottom Center: An arrow points down to a brain model titled lambda Mapped on Gray Matter. Red and yellow clusters indicate High lambda Regions, while gray areas indicate Other Voxels where lambda equals 0.

* Bottom Left: An arrow from the brain model points to a diagram of Neighboring Points Divergence, showing a red and blue dot moving apart.

* Footer: The process concludes with the text Quantifying Complexity of Brain Areas and lambda Transferred Back to G M Segmented Image.

Navigate back to Figure 1.

### Figure 2 Long description

A network diagram consisting of eleven oval nodes containing text, connected by various curved and straight lines.

At the top center is the primary node: seems like in a village, as there are other houses in the background.

Below this, moving from left to right, are three nodes:

1. the farm is located in a mountain area.

2. maybe she’s waiting for someone.

3. on the right side, there’s a girl holding a book, looking towards the road.

In the middle layer, a central node reads: seems like she’s pregnant. This node is connected to the top node and several surrounding nodes.

In the lower-middle section, there is a node: she’s holding her abdomen.

At the bottom of the graph, from left to right, are four final nodes:

1. it seems that they are gonna plough the fields.

2. it seems to be a picture about life on a farm.

3. a man is holding his horse in the background.

4. the woman is leaning onto the tree on the left side.

Lines connect these nodes in a non-linear fashion, for example, the node about the farm being in a mountain area is connected to the node about life on a farm and the node about ploughing fields. The node regarding the girl holding a book is connected to the nodes about waiting for someone, the woman leaning on a tree, and the man holding a horse.

Navigate back to Figure 2.

### Table 2 Long description

The table contains four columns: Variable, P S x Mean (std), H C Mean (std), and F D R-corrected p-value. Significant results (p < 0.05) are marked with an asterisk.

General Variables:

* SentNum: P S x 11.15 (3.14), H C 12.7 (2.58), p = 0.02.

* SentLen: P S x 11.42 (2.01), H C 11.93 (1.99), p = 0.19.

* WordNum: P S x 122.96 (36.36), H C 143.26 (29.8), p = 0.001.

* WordTTR: P S x 0.88 (0.09), H C 0.86 (0.16), p = 0.47.

* LexCatNum: P S x 462 (237), H C 583.6 (246.3), p = 0.012.

* LexCatTTR: P S x 0.72 (0.16), H C 0.72 (0.12), p = 0.95.

* Phrases: P S x 1.21 (0.05), H C 1.19 (0.044), p = 0.004.

* Depth: P S x 8.65 (1.35), H C 8.80 (1.21), p = 0.54.

* Word_PPL: P S x 6.95 (1.82), H C 5.63 (0.89), p < 0.001.

* CLIP: P S x 0.17 (0.012), H C 0.18 (0.012), p = 0.004.

Fast text model:

* LSim: P S x 0.24 (0.03), H C 0.23 (0.02), p = 0.03.

* CSim: P S x 0.47 (0.03), H C 0.47 (0.02), p = 0.047.

* CC: P S x 0.49 (0.05), H C 0.46 (0.03), p < 0.001.

* Cluster: P S x 0.49 (0.05), H C 0.49 (0.04), p = 0.39.

Sentence transformers:

* LSim: P S x 0.71 (0.11), H C 0.73 (0.02), p = 0.03.

* CSim: P S x 0.83 (0.12), H C 0.86 (0.01), p = 0.02.

* CC: P S x 0.65 (0.09), H C 0.66 (0.04), p = 0.76.

* Cluster: P S x 0.62 (0.12), H C 0.63 (0.08), p = 0.45.

B E R T:

* LSim: P S x 0.49 (0.07), H C 0.51 (0.02), p = 0.003.

* CSim: P S x 0.55 (0.08), H C 0.58 (0.01), p = 0.008.

* CC: P S x 0.36 (0.05), H C 0.37 (0.02), p = 0.51.

* Cluster: P S x 0.42 (0.06), H C 0.43 (0.02), p = 0.043.

Navigate back to Table 2.

### Figure 3 Long description

A legend at the top defines four color-coded categories. Red represents the Hold-out testing set. Green represents the Training set split into 10 folds. Purple represents the Validation set to select the winner model. Light blue represents the Training set.

Below the legend are two 10 by 10 grids.

The left grid is labeled 10 folds on the top and 10 repetitions on the left vertical axis. It consists of green squares with a diagonal line of red squares running from the top-left to the bottom-right.

A curved arrow points from the top of the right grid back to the top of the left grid.

The right grid is also labeled 10 folds on the top and 10 repetitions on the left vertical axis. It consists of light blue squares with a diagonal line of purple squares running from the top-left to the bottom-right. A horizontal white arrow points from the top-left of this grid toward the right.

To the right of the grids is a rectangular box containing the text. T-test. 200 ranked voxels selected and validated.

Navigate back to Figure 3.

### Figure 4 Long description

A three-panel figure labeled a, b, and c.

Panels a and b: Two sets of brain heatmaps showing axial, sagittal, and coronal views. Panel a displays distances from the center of mass with a smooth color gradient from blue to red. Panel b displays weighted distances with a more granular, intensity-dependent color distribution.

Panel c: A large box plot titled Skewness of Weighted Distances per R O I per Group.

- The Y-axis is labeled Skewness, ranging from 0 to 120.

- The X-axis is labeled Region, containing over 100 specific anatomical labels including Precentral L and R, Frontal regions, Cingulate, Occipital, Parietal, Temporal, and Thalamic regions.

- The legend identifies two groups: H C in blue and P S x in orange.

- Data Trend: The skewness values are highest in the frontal regions on the left side of the graph, reaching values above 100. The values generally decrease as the regions move toward the right of the axis, with the Thalamic regions (Thal) showing the lowest skewness and least variance, clustered near zero. Most regions show similar distributions between the H C and P S x groups, with overlapping interquartile ranges and numerous outliers represented by small circles above the whiskers.

Navigate back to Figure 4.

### Figure 5 Long description

The image consists of two main panels, A and B, set against a black background.

Panel A (H C group) and Panel B (P S x group) both feature a grid of 25 axial brain slices. These slices are arranged in five rows of five, labeled with numerical coordinates ranging from minus 55 at the top left to plus 60 at the bottom right. Overlaid on the gray-scale anatomical structures are blue and yellow-red heatmaps representing lambda values.

Below the axial grids in each panel are three larger anatomical projections of the brain: a lateral view, a posterior view, and a superior view.

* In Panel A, the heatmap shows moderate intensity, with a color bar scale ranging from 0.00 (blue) to 1.60 (red). The highest concentrations of red and yellow are visible in the posterior and lateral regions of the brain projections.

* In Panel B, the heatmap shows a similar distribution but with a different intensity scale. The color bar for Panel B ranges from 0.00 (blue) to 1.20 (red). The red and yellow clusters in the posterior and lateral projections appear more concentrated and widespread compared to Panel A, despite the lower maximum scale value.

Navigate back to Figure 5.

### Table 3 Long description

The table maps brain regions to six variables: P A N S S Global, P A N S S Negative, P A N S S Positive, S O F A S, Word P P L, and Word T T R. Symbols used: a green plus sign for positive associations, a red minus sign for negative associations, and a white circle for regions with both positive and negative voxels.

* Calcarine L: Negative for P A N S S Global and Word P P L; Mixed for P A N S S Negative, Positive, and S O F A S.

* Cuneus R, Lingual L, Occipital Mid L, Fusiform L, Temporal Mid L, Temporal Inf L, Cerebellum Crus 1 L and R, Cerebellum Crus 2 L, Cerebellum 9 L: Mixed across all variables except Word T T R.

* Occipital Inf L and R: Positive for P A N S S Global and S O F A S; Negative for P A N S S Negative, Positive, and Word P P L.

* Cerebellum 6 L and 8 R: Negative for P A N S S Global, Negative, Positive, and Word P P L; Positive for S O F A S.

* Cerebellum 9 R: Mixed for most; Positive for Word T T R.

* Cingulum Post L: Positive for P A N S S Global, Positive, and Word P P L; Mixed for P A N S S Negative and S O F A S.

* Calcarine R, Lingual R, Occipital Sup L and R, Occipital Mid R, SupraMarginal L, Angular L, Precuneus L, Temporal Sup L, Temporal Mid R: Positive for P A N S S Global, Negative, Positive, and Word P P L; Negative for S O F A S.

* Cuneus L: Positive for P A N S S Global, Negative, Positive, and Word P P L; Mixed for S O F A S.

* Cerebellum 6 R, 7b L, 8 L: Positive for P A N S S Global, Positive, and Word P P L; Mixed for P A N S S Negative and S O F A S (except 7b L which is Negative for S O F A S).

* Vermis 8: Positive for P A N S S Global, Negative, Positive, and Word P P L; Negative for S O F A S.

* Vermis 9: Positive for P A N S S Global, Negative, and Positive; Negative for S O F A S.

* Angular R: Positive for Word T T R only.

Navigate back to Table 3.

### Figure 6 Long description

The Y-axis represents normalized lambda values ranging from negative 2,000 to 10,000. The X-axis lists numerous brain regions with specific numerical codes. A legend at the top identifies two groups: Normalized Lambda P S x in blue and Normalized Lambda H C in orange.

Data trends from left to right:

* Precentral L 2001: P S x is approximately 3,000; H C is approximately 5,500.

* Frontal Mid Orb R 2212: P S x shows a significant peak at approximately 4,500 while H C is near zero.

* Frontal Inf Orb R 2322: P S x is approximately 4,000.

* Rolandic Oper R 2332: H C shows a major peak at approximately 7,500.

* Caudate L 7001: P S x shows a distinct peak at approximately 2,700.

* Frontal Sup Medial L 2601: H C shows the highest peak in the dataset, reaching approximately 9,000.

* Most other regions, including various Cingulum, Occipital, Parietal, and Cerebellum areas, show values near zero or slightly negative for both groups.

Navigate back to Figure 6.

### Figure 7 Long description

A multi-panel anatomical visualization consisting of 24 axial brain slices arranged in a five-by-five grid with the bottom-right corner occupied by summary diagrams.

* Axial Slices: The slices are labeled with Z-axis coordinates ranging from minus 55 at the top-left to plus 60 at the bottom-center. Each slice shows a grayscale structural M R I background with blue and yellow heat map overlays indicating voxel significance. The heat map activity is most concentrated in the posterior regions, specifically the cerebellum and occipital lobes, in the slices ranging from minus 55 to plus 15.

* Surface Projections: In the bottom-right, three white-outline brain diagrams show the cumulative voxel data from lateral, posterior, and superior perspectives. The lateral view shows a high density of significant voxels in the inferior temporal and occipital regions. The posterior view shows symmetrical clusters in both cerebellar hemispheres. The superior view shows activity concentrated in the posterior parietal and occipital areas.

* Color Scale: A vertical color bar indicates the L R P contribution values, ranging from a minimum of 0.01 represented by dark blue to a maximum of 69.67 represented by dark red. Most active voxels in the slices appear in the blue to cyan range, with small focal points of yellow and red visible in the surface projections.

Navigate back to Figure 7.
