# Supplementary material for: Structural brain complexity is associated with linguistic complexity in psychosis
Source: Eur Psychiatry. 2026 Jul 21;69(1):e86. doi: 10.1192/j.eurpsy.2026.12242 (PMC13735839; doi:10.1192/j.eurpsy.2026.12242)
Supplement: Supplementary file 1 [file S0924933826122421sup001.docx]

**Supplementary Material**

**Lyapunov Exponent**

One of the most well-known method for quantitative measures of chaos is the Largest-Lyapunov-Exponent (lambda)(*lyaprosen.m*).

Let $x_{i}$denote the spatial-series of the path free sorted weighted distances extracted from the brain sMRI. If it is assumed that the given spatial-series provides an observation of a dynamical system where the parameter time is replaced by the voxel intensity and distance from the centre of the mass combined.

Theorem of Takens^1^: the trajectory of the attractor of the system whose states evolves with spatial location over a state-space and predict the interactions over location between multiple voxel intensities can be described by a matrix X.

$X_{k}=\left[ x_{k},x_{k+\tau},x_{k+2\tau},\ldots,x_{k+\left( m-1 \right)\tau} \right]$

After the state space reconstruction, the lambda can be defined using the following equation: $\delta\left( t \right)=\delta(0)e^{\lambda t}$, where δ is the distance of the points $X_{k}$in the location k=t or k=0.

The location is considered at the state-space, meaning that that we create a space defined by 2 parameters the embedding dimension and the time delay. In the state-space these parameters calculate the number of the voxels to consider and the location of these voxels. Lambda measures how the distances diverge in the state-space, regarding the distances across all voxels selected, and thus is a ‘path-free’ measurement.

**Linguistic Features**

The correlation matrix shows that some associations are indeed present, as expected for features sampling related aspects of language, but highly redundant correlations are limited to a relatively small number of variable pairs rather than being pervasive across the full feature set. Moreover, several of the more strongly correlated pairs remain theoretically distinct and were retained for interpretability. For example, sentence length and syntactic depth are naturally related, but they capture different aspects of syntactic organization, while sentence-level closeness centrality reflects the global integration of the semantic graph rather than a simple quantitative count. Importantly, our main analyses were not based on entering all linguistic features simultaneously into a single multivariable inferential model. Thus, high pairwise correlations do not in themselves imply problematic multicollinearity in the usual regression sense. Because the goal of the study was to characterize interpretable linguistic differences across multiple domains rather than to derive a maximally compressed latent feature space, we did not adopt PCA as the primary analytical strategy.

(1) Takens, F. Detecting strange attractors in turbulence. In *Dynamical Systems and Turbulence, Warwick 1980*, Berlin, Heidelberg, 1981//, 1981; Rand, D., Young, L.-S., Eds.; Springer Berlin Heidelberg: pp 366-381.
